# Syntactic complexity differentially affects auditory sentence comprehension performance for individuals with age-related hearing loss

**DOI:** 10.3389/fpsyg.2023.1264994

**Published:** 2023-10-27

**Authors:** Junyoung Shin, Shinhee Noh, Jimin Park, Jee Eun Sung

**Affiliations:** Department of Communication Disorders, Ewha Womans University, Seoul, Republic of Korea

**Keywords:** age-related hearing loss, auditory sentence comprehension, syntactic complexity, working memory, listening effort

## Abstract

**Objectives:**

This study examined whether older adults with hearing loss (HL) experience greater difficulties in auditory sentence comprehension compared to those with typical-hearing (TH) when the linguistic burdens of syntactic complexity were systematically manipulated by varying either the sentence type (active vs. passive) or sentence length (3- vs. 4-phrases).

**Methods:**

A total of 22 individuals with HL and 24 controls participated in the study, completing sentence comprehension test (SCT), standardized memory assessments, and pure-tone audiometry tests. Generalized linear mixed effects models were employed to compare the effects of sentence type and length on SCT accuracy, while Pearson correlation coefficients were conducted to explore the relationships between SCT accuracy and other factors. Additionally, stepwise regression analyses were employed to identify memory-related predictors of sentence comprehension ability.

**Results:**

Older adults with HL exhibited poorer performance on passive sentences than on active sentences compared to controls, while the sentence length was controlled. Greater difficulties on passive sentences were linked to working memory capacity, emerging as the most significant predictor for the comprehension of passive sentences among participants with HL.

**Conclusion:**

Our findings contribute to the understanding of the linguistic-cognitive deficits linked to age-related hearing loss by demonstrating its detrimental impact on the processing of passive sentences. Cognitively healthy adults with hearing difficulties may face challenges in comprehending syntactically more complex sentences that require higher computational demands, particularly in working memory allocation.

## 1. Introduction

Age-related hearing loss is a progressive and chronic health condition that negatively affects a considerable proportion of the population (Ciorba et al., 2012)—approximately one-third of adults aged 65 years or older (World Health Organization, 2018). In recent years, a growing body of research has indicated that age-related declines in hearing may also be associated with cognitive decline (Lin et al., 2011; Humes et al., 2013). This potential link between age-related hearing loss and the cognitive demands faced by older adults may lead to declines in cognitive function (Cruickshanks et al., 1998). Furthermore, hearing-impaired older populations have a higher risk of developing dementia compared to their counterparts with typical hearing (Panza et al., 2015; Livingston et al., 2020).

Over the past decade, studies have demonstrated that the *listening effort* individuals with HL must devote to hearing and comprehending speech may burden their cognitive resources (Dubno et al., 1984; Rabbitt, 1991). The process by which a listener comprehends speech involves perceiving and analyzing the acoustic-phonetic information of the auditory stimulus and using lexical knowledge, semantic context, and syntactic knowledge (Davis and Johnsrude, 2007; Hickok and Poeppel, 2007). Rabbitt’s “effortfulness hypothesis,” initially proposed in 1968, posits that when incoming sensory signals are distorted in suboptimal listening conditions (e.g., background noise or hearing loss), listeners must devote more effort to early-stage perceptual processes. This increased effort allocation results in a reduction of cognitive resources available for encoding information. Rabbitt (1991) also demonstrated that older adults exhibited significantly improved recall when presented with word lists visually, in contrast to auditory presentation, particularly when compared to younger adults. The heightened allocation of cognitive resources during listening in challenging hearing conditions is regarded as effortful, a concept that has consistently received support in subsequent research (Sweller, 1988; Murphy et al., 2000; Pichora-Fuller, 2003; Wingfield et al., 2005; Tun et al., 2009; Rönnberg et al., 2013; Peelle, 2018).

The recent approach within the Framework for Understanding Effortful Listening (FUEL) by Pichora-Fuller et al. (2016) has advanced this concept that an additional allocation of cognitive resources might be required for individuals with HL to effectively comprehend and respond to the auditory stimuli they encounter. While Rabbitt’s hypothesis primarily focuses on the reduction of cognitive resources for encoding auditory stimuli, the FUEL model extends its scope beyond hearing difficulties alone, considering various factors, such as mental effort, fatigue, and more, which contribute to the fluctuations in listening effort (Pichora-Fuller et al., 2016). Indeed, Pichora-Fuller and her colleagues have introduced a three-dimensional framework (comprising effort, motivation, and demands) to illustrate how listening effort varies based on task demands and individual motivation. This framework underscores that listening effort depends on factors beyond hearing difficulties alone. It enables us to visualize differences among individuals, variations within individuals across different conditions, and fluctuations in effort due to changes in task demands and motivation during complex tasks.

Nonetheless, ongoing efforts to explore its potential link have often encountered inconclusive outcomes (Lindenberger and Baltes, 1994). Several studies have found a correlation between hearing impairments and poor performance on cognitive tasks (Baltes and Lindenberger, 1997; Valentijn et al., 2005; Dupuis et al., 2015; Harrison Bush et al., 2015; Yuan et al., 2018), while others have shown no significant association (Gennis, 1991; Lyxell et al., 2003; Zekveld et al., 2007; Lin et al., 2013; Wong et al., 2014). Notably, Wong et al. (2019) observed no significant differences in memory and attention task performance between old adults with and without HL when administered under visual conditions using the Hopkins Verbal Learning Test. On the other hand, McCoy et al. (2005) found that individuals with HL had lower recall abilities than their age-matched peers without HL in a word recall task, despite identifying the correct words through perceptual checks. These research efforts, taken together, suggest that exclusively analyzing a single domain of cognitive task performance may not sufficiently capture the potential cognitive challenges associated with HL, highlighting the need for further research to establish a robust methodological framework for addressing the intricate interplay between HL and cognitive resources.

Sentence comprehension is a cognitively demanding task that requires the engagement of multiple dimensions of cognitive processes (Just and Carpenter, 1992). In particular, greater processing demands, such as in syntactically complex sentences, necessitate the engagement of several high-level cognitive processes including language comprehension, memory recall, and attention control; this adds to the cognitive load for older adults (Just and Carpenter, 1992). Given that multiple complex cognitive abilities are involved in sentence processing tasks, the fact that sentence comprehension abilities decline with age is unsurprising. In older individuals with even unimpaired hearing, Rönnberg et al. (2021) highlighted that slight hearing loss can exacerbate a decline in the comprehension of syntactically more complex sentences (Ayasse et al., 2019; Rudner et al., 2019).

The sentence-picture matching paradigm is widely used to measure age-related changes in individuals’ sentence processing abilities, allowing for the analysis of syntactic structures and comprehension of sentence meaning by incorporating both syntax and semantics (Sung et al., 2021). Aging-related effects on sentence comprehension abilities can be more sensitively detected when sentences are semantically reversible and carry greater syntactic computation demands (Sung et al., 2020). Several studies have demonstrated that sentence comprehension tasks as a critical linguistic marker to reveal subtle language processing changes that may signal early cognitive decline and identify individuals with Mild Cognitive Impairment (MCI) or those at-risk in the older population (Fallon et al., 2006; Peelle, 2018; Sung et al., 2020).

The current study examines whether older adults with HL have greater difficulties processing sentences than those who have normal hearing abilities by systematically manipulating syntactic complexity: (1) computational loads: *sentence type* and (2) storage loads: *sentence length*. We analyzed the effects of *sentence type* by comparing active and passive structures while controlling for sentence length. For example, the passive sentence “*The cat was chased by the dog*” contains increased syntactic complexity, as the theme of the action is moved to the subject position and a by-phrase is used to identify the agent (Grodzinsky, 1984). Grodzinsky’s trace deletion hypothesis posits that in passive sentences, when a theme moves to the subject position, it leaves a trace in its original position. It has been reported that English-speaking agrammatic speakers often find it challenging in processing this trace left by the theme’s movement and tend to rely on word order to discern thematic roles in passive sentences. This syntactic movement has led to claims that passive sentences require greater processing resources (Caplan et al., 1985, 2007). Numerous studies across different languages have shown that difficulties in processing passive sentences are not limited to clinical populations but also encompass older adults (Obler et al., 1985; Dąbrowska and Street, 2006; Yokoyama et al., 2006; Sung, 2015a; Sung et al., 2017).

In the landscape of linguistic research, Korean offers a unique contribution to existing literature on passive sentence processing. While most of the evidence comes from SVO (Subject-Verb-Object) languages like English, Korean follows SOV with a relative flexibility to scramble the word order that allows both SOV and OSV structures. This flexibility is enabled by a case marking system that denotes the structural functions of linguistic units within a sentence. The passivization process in Korean bears similarities to that in English, given that the noun phrase (NP) of the theme is moved to the subject position and a by-phrase is created for the agent, accompanied by the morphological inflections of the verbs. However, a distinguishing feature of Korean is that its passive sentences maintain the same length as their active counterparts due to its case marking systems. This unique attribute allows for a direct comparison of the computational loads between active and passive structures, with sentence length held constant. Studies have indicated that Korean speakers demonstrated greater difficulties in processing passive than active sentences across diverse groups, including older adults (Sung, 2015a,b; Sung et al., 2017) and clinical populations, such as those with aphasia (Sung et al., 2018) and mild cognitive impairment (MCI) (Sung et al., 2020).

**Figure 1:**
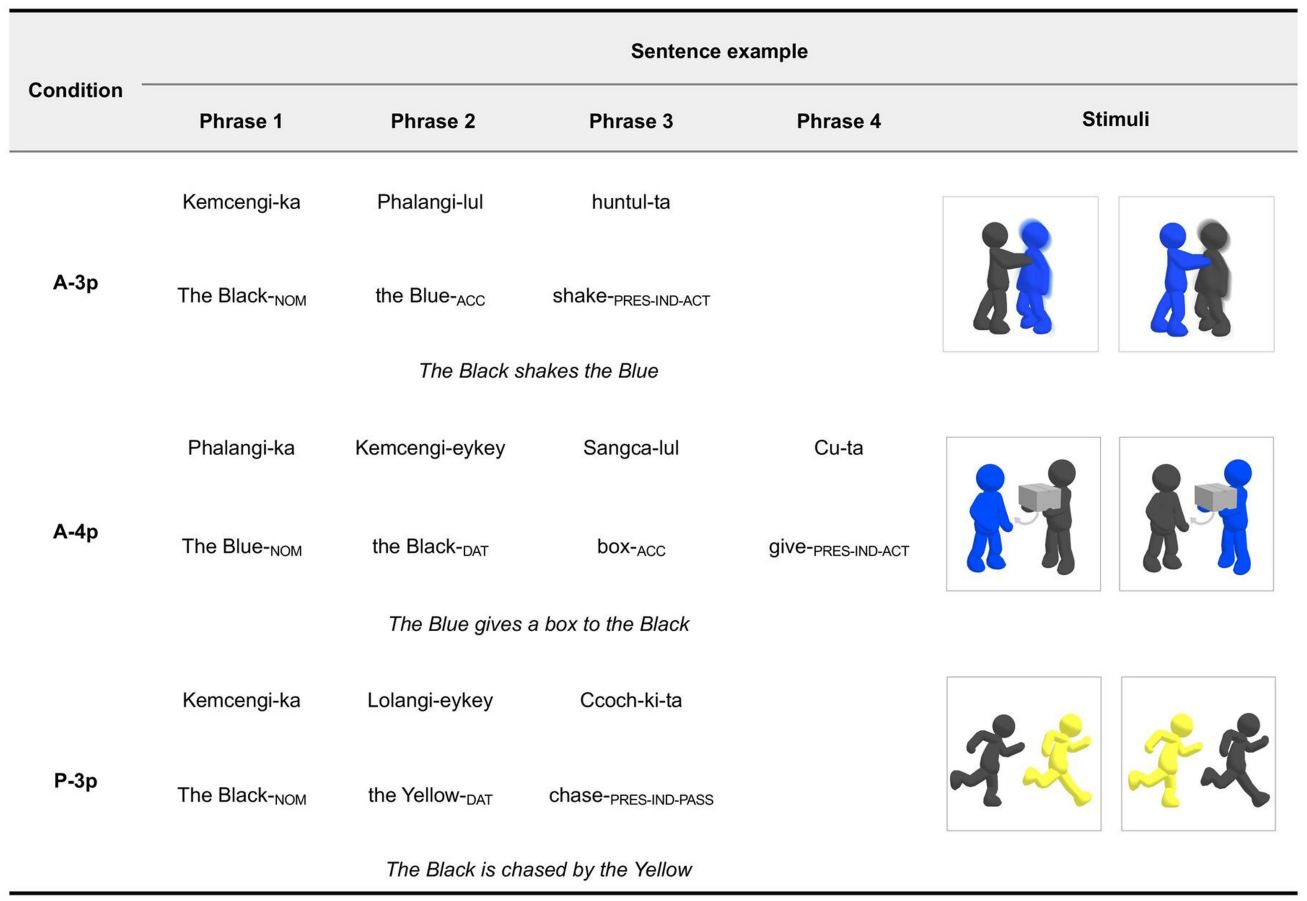
Example of the sentence stimuli in the sentence comprehension task (Sung, 2015b). A-3p, Active with 3 phrases; A-4p, Active with 4 phrases; P-3p, Passive with 3 phrases; NOM, nominative case-marker; ACC, accusative case-marker; PRES, present tense suffix; IND, indicative mood suffix; ACT, active suffix; DAT, dative case-marker; PASS, passive suffix.

Another factor that we manipulated is *sentence length,* by varying the number of phrases in active sentences. Specifically, we manipulated the number of phrases with different argument structures associated with the verbs in given sentences. In linguistics, the term “argument” refers to the elements that represent the targets of the syntactic relationships conveyed by verbs (Si et al., 2000). An increase in the number of argument structures within a verb leads to longer sentence structures, as indicated by an increased number of phrases (Herlofsky and Edmonds, 2012). We compared active sentences with 3-phrases (e.g., The Black shakes the Blue) to those with 4-phrases (e.g., The Blue gives a box to the Black) as illustrated in Figure 1. Previous studies employing this methodological framework have generated evidence of the comparative effects of sentence length on auditory sentence comprehension, particularly in aging populations with cognitive disorders (dementia of the Alzheimer type: Small et al., 1991; Rochon et al., 1994). These studies have demonstrated improved auditory sentence comprehension when shorter sentences are presented. The differences in performance based on sentence length have been attributed to the increased number of propositions conveying information about events, situations, or relationships in the sentences. According to the *propositional theory* postulated by Kintsch (1974), propositions represent the meaning that is capable of being stored and recalled from the memory system. As the number of propositions increases, listeners experience cognitive overload in interpreting the underlying messages within the sentences (Kintsch, 1974; Bayles and Tomoeda, 2007).

Since working memory was identified as a cognitive mechanism involved in storing and computing information in the short term (Baddeley and Hitch, 1974), studies consistently demonstrate a close link between sentence comprehension abilities and working memory capacity (Just and Carpenter, 1992; Montgomery et al., 2008). Working memory plays a crucial role in the process of language comprehension, allowing individuals to connect linguistic elements, such as words and phrases, and analyze the structural aspects of language (Marton and Schwartz, 2003). The Ease of Language Understanding (ELU) proposed by Rönnberg (2003) and Rönnberg et al. (2008) posits the critical role of working memory in language understanding, especially in challenging listening conditions (e.g., hearing impairment and background noise) or when language processing imposes a cognitive load. Within the framework of ELU, individuals with limited working memory capacity may encounter challenges in comprehending and processing language, especially when dealing with complex or demanding sentences. Wendt et al. (2015) examined the association between sentence processing and cognitive abilities among adults with HL, finding a significant correlation between their performance on word span tests and processing duration for ambiguous sentences with non-canonical word order structures among individuals with HL. DeCaro et al. (2016) investigated the impact of age-related demographic factors, including age, hearing status, and memory capacity on sentence comprehension in older adults with HL. The findings revealed that reading span, a measure of working memory, emerged as a robust predictor of comprehension accuracy in all conditions for hearing-impaired individuals.

Another line of research has suggested that semantic memory plays a role in integrating new semantic information into one’s existing knowledge during sentence processing (Federmeier et al., 2010). Unlike working memory, which derives from the short-term memory system, semantic memory is a part of the long-term memory system. It was originally defined by Tulving (1972) as a *mental thesaurus*, which is organized one’s knowledge about words, their meanings, and the rules for manipulating these elements, refers to general knowledge (Rönnberg et al., 2013). Previous studies have reported that individuals with semantic memory impairments have difficulties comprehending sentences with increased semantic information (Martin and Romani, 1994; Martin and He, 2004). A recent study by Horne et al. (2022) found that in adults with chronic aphasia, semantic memory has a significant independent contribution to comprehension of relative clause sentences with a passive construction.

Semantic memory is often assessed using a verbal learning test (Farrow et al., 2010), which consists of both immediate and delayed recall sections separated by a 20-min interval. In the immediate recall section, participants are presented with a series of words to encode, and then asked to recall them immediately after. In the delayed recall section, participants are asked to recall the words after a 20- min gap. Given this paradigm, the delayed recall part of the test primarily taps into long-term memory capabilities, as it specifically assesses an individual’s ability to retrieve information over a span where memory decay occurs between the immediate and delayed recall phrases. Few studies have investigated the effects of hearing impairments on word recall performance. Tun et al. (2009) pointed out that older adults with even mild sensory impairments may exhibit significantly reduced performance in word-list recall tasks due to the increased cognitive effort required for successful perception. Furthermore, Rönnberg et al. (2011) observed a significant negative correlation between hearing thresholds for both ears and word recall performance in older adults with hearing loss who used hearing aids. Nevertheless, the potential connection between semantic memory and sentence-level processing in this population remains understudied.

In this study, we investigated whether older adults with HL had difficulties in the sentence comprehension task by systematically varying the syntactic burdens either from the computational loads (sentence type: passive vs. active) or storage buffer (sentence length: 3- vs. 4-phrase). Specifically, we examined whether older adults with HL perform differentially worse on sentence types with greater resources (passive > active) and sentences with more linguistic units (4-phrase >3-phrase) than their controls with normal hearing. We also explored whether the ability to comprehend sentences with varying levels of syntactic processing demands is associated with working memory, semantic memory, and hearing acuity, aiming to identify the most influential predictors of sentence comprehension abilities within each group.

## 2. Method

### 2.1. Participants

A total of 46 Korean-speaking older adults participated in the study. All participants performed within normal ranges (age- and education-adjusted scores ≥16%ile) on the Korean Mini-Mental State Examination (K-MMSE; Kang, 2006), the Digit Span test, the Seoul Verbal Learning Test (SVLT; Kang and Na, 2003) from a standardized Seoul Neuropsychological Screening Battery-II (SNSB-II; Kang et al., 2012), and a short version of the Geriatric Depression Scale (Bae and Cho, 2004; < 8 out of 15). According to the self-reported information they provided, none of the participants had any vision impairments, histories of brain injuries. The Ewha Woman’s University Institutional Review Board (No. 2022–0112) authorized this study, and we acquired written consent from all participants.

Participants were assigned to either the *Typical-hearing* (TH) or *Hearing Loss* (HL) groups based on their mean pure-tone average (PTA): (i) 24 participants (58% females) comprised the “typical-hearing” TH group, and (ii) 22 participants (50% females) comprised the “hearing loss” HL group.

The HL group was selected with the hearing loss greater than 35 dB HL with the mean PTA for both ears at 0.5, 1, 2, and 4 kHz, respectively (Abutan et al., 1993), as a person with moderate and moderate-to-severe hearing loss (World Health Organization, 1991), and the difference in the average hearing threshold level of both ears within 10 dB HL when using a pure-tone audiometer (Grason-Stadler GSI 18). In the case of the TH group, the average hearing in both ears was within the 32 dB hearing level at 0.5 to 4 kHz, respectively (WHO, 1991). Regarding the HL group’s mean PTA thresholds for each frequency: left ear – 0.5KHz (*M*: 41.13, SD: 9.76), 1KHz (*M*: 41.81, SD: 12.75), 2KHz (*M*: 50, SD: 10.11), 4KHz (*M*: 60.22, SD: 4.64); right ear – 0.5KHz (*M*: 40.22, SD: 9.59), 1KHz (*M*: 41.36, SD: 11.49), 2KHz (*M*: 47.72, SD: 10.19), 4KHz (*M*: 54.77, SD: 8.32). In the TH group, the mean PTA thresholds were as follows: left ear – 0.5KHz (*M*: 23.75, SD: 6.16), 1KHz (*M*: 22.29, SD: 6.45), 2KHz (*M*: 25.83, SD: 6.06), 4KHz (*M*: 33.54, SD: 11.03); right ear – 0.5KHz (*M*: 23.33, SD: 6.71), 1KHz (*M*: 21.66, SD: 5.52), 2KHz (*M*: 25.62, SD: 6.96), 4KHz (*M*: 35.83, SD: 11.42).

We conducted an independent sample *t*-test at the 0.05 significance level to see if there were statistically significant between-group differences in age and years of education, K-MMSE, SVLT, and DST. The test revealed no significant difference in age (*p* = 0.726), education level (*p* = 0.605), K-MMSE (*p* = 0.704), SVLT immediate recall (*p* = 0.753), SVLT delayed recall (*p* = 0.436), Digit forward (*p* = 0.232), and Digit backward (*p* = 0.093). Test statistics and corresponding *value of p*s for each descriptive measure in two groups are presented in Table 1.

**Table 1:** Descriptive information for participants in each group.

Characteristic	TH group^a^	HL group^b^	Test statistics	Value of *p*
Gender (male: female)	10:14	11:11	-	-
Age (year)	71.50 (5.86) 65–84	72.09 (6.32) 65–88	0.123	0.726
Years of education	10.50 (4.36) 6–18	9.86 (3.87) 6–16	0.275	0.605
K-MMSE	28.04 (1.63) 24–30	28.23 (1.66) 24–30	0.146	0.704
Mean PTA	26 (5) 15–32	46 (9) 35–65	-	-
SVLT				
Immediate recall	19.21 (3.67) 15–25	19.55 (3.56) 15–25	0.100	0.753
Delayed recall	5.42 (1.44) 4–8	5.86 (2.27) 4–11	0.621	0.436
Digit span				
Digit forward	6.38 (1.38) 4–9	5.91 (1.23) 4–8	1.468	0.232
Digit backward	4.29 (0.85) 3–7	3.86 (0.83) 3–6	2.941	0.093

Values are presented as means (SD). ^a^*n* = 24, ^b^*n* = 22. K-MMSE, Korean-Mini Mental State Examination (Kang, 2006); SVLT, Seoul Verbal Learning Test (SVLT; Kang and Na, 2003); Digit Span Test (Kang et al., 2012); Mean PTA, Mean pure-tone average (dB); TH, Typical-hearing; HL, Hearing loss. *p < 0.05; **p < 0.01.

### 2.2. Materials

#### 2.2.1. Sentence comprehension test

We conducted a sentence comprehension test (SCT) (Sung, 2015b) designed to examine the influence of syntactic structure on sentence comprehension with semantically reversible sentences. Each thematic role within a sentence is represented by incorporating human-like figures with three distinct colors (the yellow, the black, the blue) in these pictograms. Consequently, these humanized representations reduce the influence of top-down semantic processing on syntactic comprehension.

It comprised 36 total items with 12 items for each of the three sentence conditions [active sentence with three phrases (A-3p), active sentence with four phrases (A-4p), passive sentence with three phrases (P-3p)]. For each structure, the word order of canonicity was counterbalanced.

Each item was displayed using a sentence-picture matching paradigm that presented pictures of the target and its syntactic foil (Figure 1). Participants were instructed to select the picture that matched the verbally presented sentence. Before the main test, participants complete four practice trials to ensure the accuracy of their color perceptions of the stimuli. If participants requested a sentence repetition, they were given only one repetition of the sentence, and their final response was documented. The responses for each item were coded as 1 for correct and 0 for incorrect responses.

### 2.3. Memory measures

#### 2.3.1. Digit Span test

We utilized the Digit Span (DS) task from a standardized neuropsychological assessment (SNSB-II; Kang et al., 2012) and followed the established procedures. It consists of two components: Digit forward and Digit backward. In the Digit forward task, participants were presented with a series of orally spoken digits, ranging from 3 to 9 digits, and they had to repeat them aloud. In the Digit backward task, participants were again presented with a series of orally spoken digits, ranging from 2 to 8 digits, and their task was to repeat the digits in reverse order. Each span consisted of 2 items, and participants progressed to the next span if they correctly recalled the first item. The administration terminated if participants failed to recall both items accurately. The longest sequence of digits successfully recalled, in either correct or reverse order, represented participants’ Digit forward and Digit backward spans. We calculated an index of participants’ WM capacity as the mean value obtained by averaging the Digit forward and Digit backward spans.

#### 2.3.2. Seoul Verbal Learning Test

We administered the SVLT (Kang and Na, 2003) from SNSB-II (Kang et al., 2012), following the established procedures. The test comprises two subtests: immediate recall (IR) task and delayed recall (DR) task, involving a total of 12 words. These words are categorized into three semantic categories, flowers, kitchen utensils, and stationery, with each category containing four words. In the IR, participants are required to recall the 12 randomized-order words right after they are presented auditorily, and this procedure is repeated three times. Following a 20-min interval, participants are asked to recall the words from the IR without any auditory presentation. Regardless of the categories, the examiner scored the total number of correctly recalled words, with a maximum score of 36 (12 items × 3 times) for the IR involving three trials and 12 for the DR.

### 2.4. Experimental procedures

We individually tested participants in a separate and sound-attenuated setting, and administered the memory measures in compliance with established standardized protocols. Following the tests, we conducted the SCT as described by Sung (2015a). First, we introduced the characters Yellow, Blue, and Black in the picture and the actions that they do. For each sentence, we presented two pictures, one representing the target and the other the foil. Participants were then instructed to respond by pointing at the target picture after hearing the sentence delivered *via* an auditory presentation from a built-in speaker located 50 cm directly in front of them. Initially, all participants were exposed to a sound pressure level of at least 70 dB. However, in cases where participants expressed a preference for increased volume during practice trials, adjustments were made. If a participant requested to hear the sentence again, the evaluator repeated it once. One participant wore hearing aids throughout all testing phases of this study. After four practice questions and volume adjustments, participants proceeded to the main task. The entire process of the experimental procedures was recorded.

### 2.5. Inter-rater reliability

To ensure the reliability of the scored performances, inter-rater agreement was assessed for 35% of the participants. There was a 100% consensus among the three evaluators, as confirmed by their review of the recorded video.

### 2.6. Statistical analyses

We conducted two generalized linear mixed model fit by adaptive Gauss-Hermite quadrature (Stringer and Bilodeau, 2022) using the glmer function from the lme4 package (Bates et al., 2015) and lmerTest (Kuznetsova et al., 2014) in R software (R Core Team, 2020). In the first model, fixed effects included group and sentence type [A-3p vs. P-3p; reference levels: Group = HL, Sentence type = Active], while in the second model, group and sentence length was incorporated [A-3p vs. A-4p; reference levels: Group = HL, Sentence length = 3p]. For random effects, we included a random by-subject intercept in all models. Notably, adaptive Gaussian Quadrature was employed, which is only available for models with a single scalar random-effects term (Stringer and Bilodeau, 2022). We selected the by-subject random factor due to its lower AIC value (Pan, 2001; Yu and Yau, 2012), compared to by-item. Importantly, there were no significant statistical differences between the two random factor choices, whether by-subject or by-item. Pearson correlation coefficients were calculated to assess the relationships among the conditions from the SCT [A-3p, A-4p, P-3p], memory measures [DS, IR, DR] scores, and the mean PTA for each group. Further, we utilized multiple stepwise regression analysis to examine the memory-related factors predicting each group’s sentence comprehension performance.

## 3. Results

### 3.1. Sentence type effect

**Figure 2:**
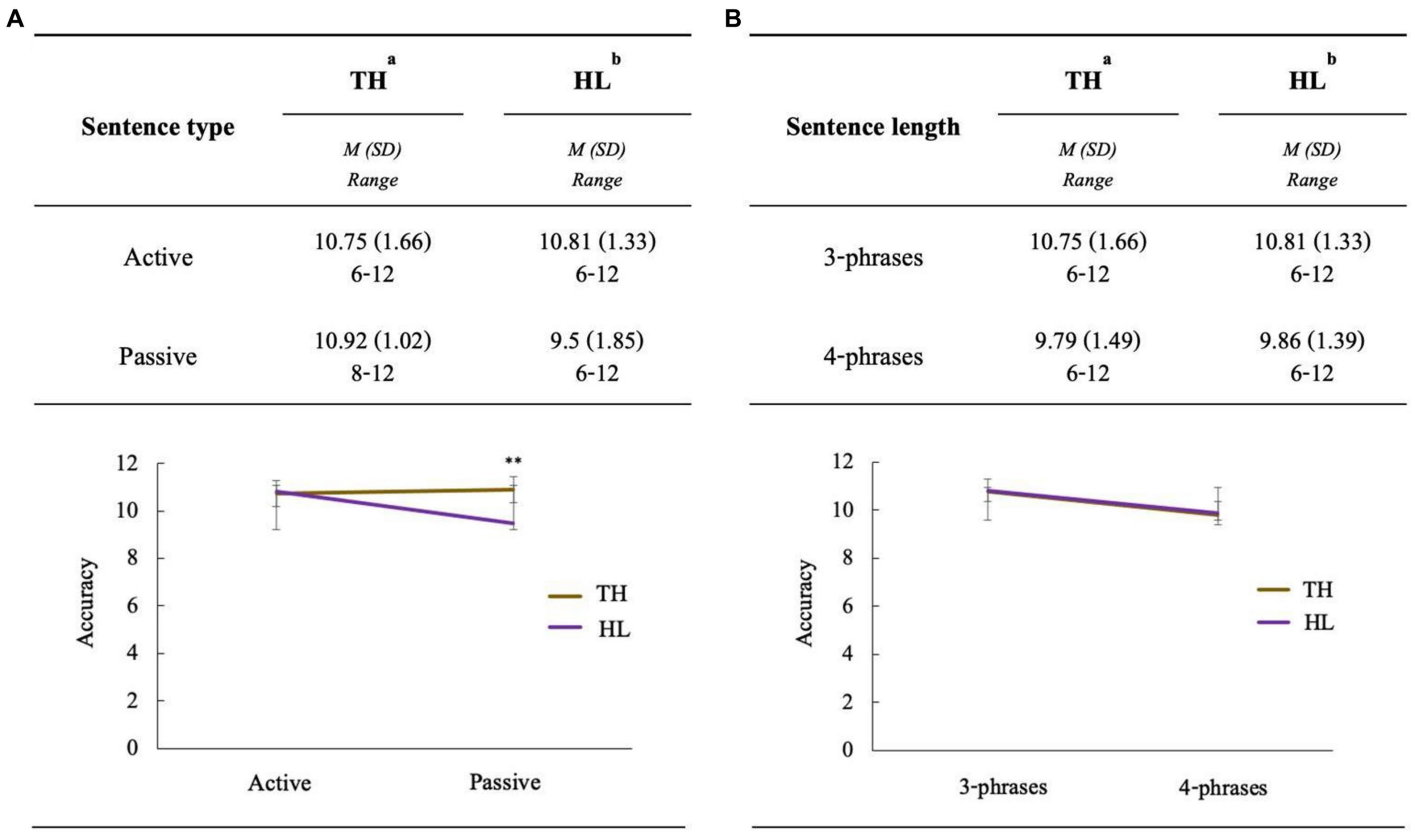
**(A)** Accuracy of the sentence comprehension test depending on sentence type for each group. **(B)** Accuracy of the sentence comprehension test depending on sentence length for each group. ^a^*n* = 24; ^b^*n* = 22; TH, Typical-hearing; HL, Hearing loss. Error bar is based on standard error. **p < 0.01.

Descriptive statistics for the SCT accuracy are presented using means and standard deviations (Figure 2A). Our analysis revealed that the although the main effect for *group* was not significant (*β* = −0.0247, *SE* = 0.3875, *z* = −0.064, *p* = 0.9491), the main effect for *sentence type* was significant (*β* = −0.9445, *SE* = 0.2658, *z* = −3.553, *p* = 0.0003), indicating that passive structures elicited more errors than active sentences. In addition, the two-way interaction between *group* and *sentence type* was significant (*β* = 1.1135, *SE* = 0.3943, *z* = 2.824, *p* = 0.0047); the HL group demonstrated differentially lower accuracy in the passive versus active structures than the TH group (Table 2; Figure 2A).

**Table 2:** Generalized linear mixed-effects regression models of sentence type effect and sentence length effect.

Fixed effects	Estimate	*SE*	*z*	Value of *p*
Sentence type				
(Intercept)	2.4445	0.2850	8.576	< 2e-16
Group	−0.0247	0.3875	−0.064	0.9491
Sentence type	−0.9445	0.2658	−3.553	0.0003***
Group x Sentence type	1.1135	0.3943	2.824	0.0047**
Sentence length				
(Intercept)	2.3889	0.2659	8.984	< 2e-16
Group	−0.0408	0.3606	−0.113	0.9099
Sentence length	−0.7226	0.2691	−2.685	0.0072**
Group x Sentence length	0.0173	0.3695	0.047	0.9625

Formula: Sentence type [Accuracy ~ Group * Sentence type + (1 | Subject)]; Sentence length [Accuracy ~ Group * Sentence length + (1 | Subject)]. SD, Standard Deviation; SE, standard error. ***p < 0.001, **p < 0.01.

### 3.2. Sentence length effect

While the main effect for *group* was not significant (*β* = −0.0408, *SE* = 0.3606, *z* = −0.113, *p* = 0.9099), the main effect for *sentence length* was significant (*β* = −0.7226, *SE* = 0.2691, *z* = −2.685, *p* = 0.0072), indicating that longer sentences with four phrases elicited more errors than shorter sentences with three phrases. Meanwhile, we found no significant interactions between the *sentence length* and *group* (*β* = 0.0173, *SE* = 0.3695, *z* = 0.047, *p* = 0.9625), as shown in Table 2 and Figure 2B.

### 3.3. Analyses of Pearson correlation coefficients

**Figure 3:**
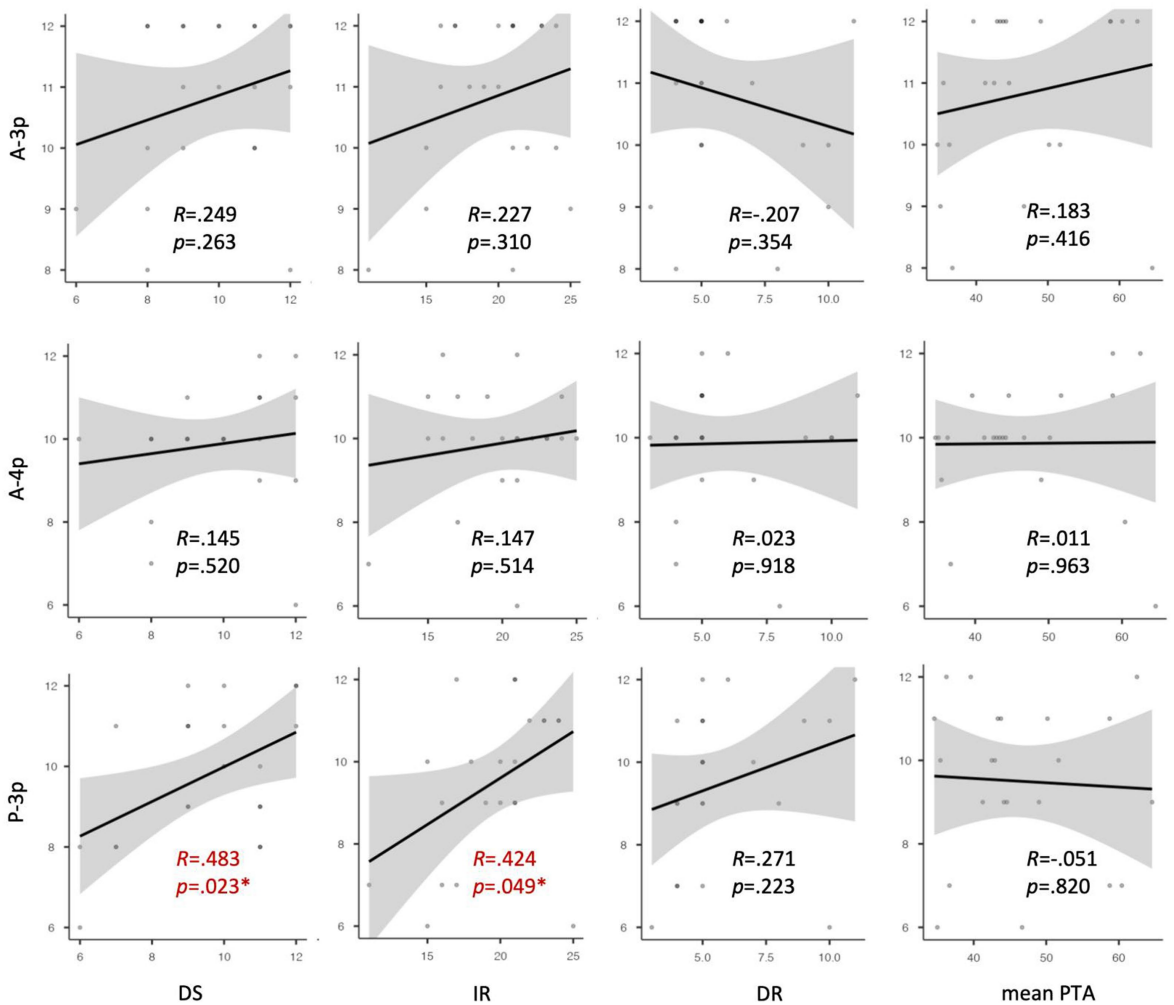
Scatter plots illustrating the relationship between sentence comprehension test performance and memory measures, as well as the mean PTA in the hearing loss group. A-3p, Active with 3 phrases; A-4p, Active with 4 phrases; P-3p, Passive with 3 phrases; DS, Digit span test; IR, Immediate recall; DR, Delayed recall; mean PTA, Mean pure-tone average.

We computed Pearson correlation coefficients among the SCT conditions [A-3p, A-4p, P-3p], and scores of memory measures [DS, IR, DR], as well as mean PTA for each group (Table 3). In the HL group, the P-3p condition was significantly correlated with both DS (*r* = 0.424, *p* = 0.023) and IR scores (*r* = 0.311, *p* = 0.049), as illustrated in Figure 3. However, A-3p did not significantly correlate with other variables, and neither did A-4p. In the TH group, there were no significant correlations between any of the SCT conditions and other variables.

**Table 3:** Pearson correlation coefficients among three sentence conditions, memory measure scores, and mean PTA for each group.

Variables	DS	IR	DR	Mean PTA
A-3p				
TH	0.325	0.030	0.262	0.045
HL	0.249	0.227	−0.207	0.183
A-4p				
TH	0.209	0.078	0.080	0.325
HL	0.145	0.147	0.023	0.011
P-3p				
TH	0.220	0.354	0.262	−0.264
HL	0.483*	0.424*	0.271	−0.051

TH, Typical-hearing group; HL, Hearing loss group; A-3p, Active with 3 phrases; A-4p, Active with 4 phrases; P-3p, Passive with 3 phrases; DS, Digit span test; IR, Immediate recall; DR, Delayed recall; Mean PTA, Mean pure- tone average. ***p < 0.001, **p < 0.01, *p < 0.05.

### 3.4. Analyses of stepwise multiple regression

Stepwise multiple regression analysis was conducted to explore which memory measure best predicts each SCT condition (A-3p, A-4p, P-3p) for each group. We included DS, IR, and DR as independent variables. In the HL group, the first model revealed that DS (*β* = 0.483. *p* = 0.023) was a significant predictor of P-3p [*F*_(1, 21)_ = 6.099, *p* = 0.023, *R*^2^ = 0.234], which explained 23.4% of the variance (Table 4). The final model revealed that DS (*β* = 0.459. *p* = 0.019) and IR (*β* = 0.396. *p* = 0.040) were significant predictors of P-3p [*F*_(2, 21)_ = 6.065, *p* = 0.009, *R*^2^ = 0.390], which explained 39.0% of the variance. Table 4 represented the beta scores of the variables in this model. However, in the TH group, none of the memory measures significantly predicted A-3p, A-4p, or P-3p.

**Table 4:** Coefficient of the stepwise multiple regression model for the predictor of the passive with three phrases in the hearing loss group.

Variables	Unstandardized coefficients		Standardized coefficients	Test statistics	Value of *p*
	*B*	SE	*β*		
Model 1					
(Constant)	4.194	2.179		1.925	0.069
DR	0.543	0.220	0.483	2.470	0.023*
Model 2					
(Constant)	0.336	2.654		0.127	0.901
DR	0.516	0.202	0.459	2.557	0.019*
IR	0.211	0.096	0.396	2.204	0.040*

The dependent variable is the passive with three phrases. SE, standard error. ^*^p < 0.05.

## 4. Discussion

We investigated whether participants with HL would experience greater difficulties processing sentences than those with TH when syntactic complexity—represented by length and type—varied. Our findings revealed that older adults with HL performed worse than TH participants on passive sentences versus active sentences when sentence length was controlled. In contrast, our manipulation of sentence length did not elicit group-based differences. Additionally, for the HL group, we found significant correlations between DS scores and accuracy on passive sentences, as well as between IR scores and passive sentences. Regression analyses confirmed that both DS and IR, with DS having a greater influence than IR (*p* = 0.019 and *p* = 0.040, respectively), significantly predicted performance on passive sentences within the HL group. This suggests that HL group’s ability to process more challenging sentence structures (e.g., passive) is more closely associated with short-term based WM capacity than long-term semantic memory component.

The current results are particularly intriguing in that the HL group was differentially affected by the manipulation of the computational loads on the sentence type but not by sentence length. Our findings are consistent with the previous hypotheses on listening efforts (Rönnberg et al., 2008; Pichora-Fuller et al., 2016; Peelle, 2018), suggesting that individuals with hearing difficulties experience degraded performance as processing demands increase, notably taxing cognitive capacity. Indeed, individuals maintained their performance even in the presence of HL, especially in syntactically less complex sentences with an active structure, due to the fact that the processing demands did not exceed their cognitive resource limit (Rönnberg, 2003). Considering that, in this experiment, we controlled for other factors unrelated to hearing acuity like age, years of education, and cognitive function between groups, it appears that the challenges observed in the HL group arise from having fewer resources available to be allocated to the more complex sentence types. This is likely due to their hearing loss, which requires greater listening effort than that of the TH group. These effects become especially pronounced when task demands exceed their capacity.

The most decreased performance on the sentence types with the highest computational load (e.g., passive) was significantly predicted by DS, followed by the IR. These results may imply that the underlying cognitive process engaged in the SCT (Sung, 2015b) predominantly relies on short-term memory system rather than long-term memory, aligning with prior aging studies that employed sentence-picture matching paradigms (Schumacher et al., 2015; Sung, 2017; Liu, 2018; Sung et al., 2020). In contrast, DR exhibited no significant correlation with performance on passive sentences and did not emerge as a significant predictor. This discrepancy can be attributed to the inherent characteristics of DR, which taps into the long-term memory component, specifically given that it measures the ability to recall words after a 20-min decay. In light of these findings, it could be interpreted that the HL group, especially under suboptimal listening conditions, needed to allocate additional working memory resources to effectively process sentences with heightened computational demands, as previously proposed in Rönnberg et al. (2008). This interpretation was further reinforced by our outcomes from the stepwise regression analysis, which align with the prior findings by DeCaro et al. (2016) that working memory capacity measured by reading span tests serves as a significant predictor of object-relative sentence comprehension in older adults with mild-to-moderate hearing loss.

What is evident from the current data is that passive constructions exert a sufficient cognitive burden, leading to performance degradation in the HL group, even though they are syntactically simpler than center-embedded construction. A couple of studies have consistently reported that older adults with HL find it challenging to understand syntactically intricate sentences, especially those containing relative clauses like subject-relative and object-relative structures (Wingfield et al., 2006; Tun et al., 2010; Amichetti et al., 2016; DeCaro et al., 2016). However, our study’s active-passive contrasts maintained an equivalent sentence length, allowing us to directly measure how listening effort changes among hearing-impaired adults due to computational load rather than storage load, especially when comprehending auditory sentences. Even though the syntactic structures used in the study are simpler than those in previous studies with center-embedded sentences containing relative clauses, it is noteworthy that our paradigm still elicited performance degradation in the HL group. We speculate that this may be because we employed semantically reversible sentences. By constraining top-town semantic processing, the SCT paradigm forced participants to rely solely on grammatical markers to fully parse a sentence (Sung, 2015b). The results suggest that even simpler structures, when tailored to reflect specific linguistic features within a constrained paradigm reflecting, can contribute to making a differential diagnosis of sentence processing difficulties in the HL group. To validate these findings, the current paradigm should be replicated across a variety of languages, both with and without case marking systems. This would confirm the hypothesis that a simpler structure with increased computational loads is a more effective predictor than the manipulation of sentence length.

In summary, our study investigated how syntactic complexity and hearing difficulties influence sentence comprehension in aging populations with hearing impairment, employing the sentence-picture matching paradigm-based SCT (Sung, 2015b). As many nations have already transitioned into aging societies, the systematic monitoring of declines in complex cognitive processing linked to age-related hearing loss becomes increasingly crucial. We recommend the adoption of this methodological framework in various linguistic contexts for future research, as it holds the potential to shed light on the potential connection between age-related hearing loss and cognitive decline.

Our study has limitations to consider: First, it should be noted that the definition of listening effort remains a topic of ongoing debate, as demonstrated in the work of DeCaro et al. (2016) and McGarrigle et al. (2014). Second, the presentation of auditory sentences *via* speakers varied based on participants’ reported comfort levels in terms of volume (dB). Although all participants were initially exposed to a minimum sound pressure level of 70 dB, some individuals requested adjustments for louder sound levels. To address potential sound level-related effects, we suggest presenting sentences binaurally through insert earphones or headphones, following the methodology employed by Ayasse et al. (2017), ensuring audibility at 25 dB above each individual’s better-ear speech reception threshold.

## Data availability statement

The datasets for this manuscript are not publicly available due to the approved ethical conditions of the study. Further inquiries can be directed to the corresponding author.

## Ethics statement

The studies involving humans were approved by Institutional Review Board of Ewha Womans University. The studies were conducted in accordance with the local legislation and institutional requirements. Written informed consent for participation in this study was provided by the participants’ legal guardians/next of kin.

## Author contributions

JS: Conceptualization, Formal analysis, Investigation, Writing – original draft, Methodology, Visualization. SN: Investigation, Writing – original draft. JP: Investigation, Writing – original draft. JES: Conceptualization, Project administration, Supervision, Writing – review & editing.
